# Impact of environmental factors and climate conditions on the occurrence of *Vibrio* and *Shewanella* infections in Norway, 2014–2018

**DOI:** 10.1186/s12889-026-27550-7

**Published:** 2026-05-04

**Authors:** Beatriz Valcarcel Salamanca, Andrew Luke King, Anne Deininger, Susanne Hyllestad, Emily Macdonald, Umaer Naseer, Ettore Amato

**Affiliations:** 1 Division of Infection Control, Norwegian Institute of Public Health (NIPH), Oslo, Norway; 2 ECDC Fellowship Programme, Field Epidemiology path (EPIET), European Centre for Disease Prevention and Control (ECDC), Stockholm, Sweden; 3 Department of Oceanography, Norwegian Institute for Water Research (NIVA), Oslo, Norway

**Keywords:** Surveillance, Vibrio, Shewanella, Seawater temperature and salinity, Climate, Early-warning, Norway

## Abstract

**Background:**

*Vibrio* and *Shewanella* spp. (VS) are climate-sensitive bacteria found in marine environments, which sometimes cause severe human infections. VS infections have risen globally, particularly in Northern Europe. In this study, we aimed to describe the epidemiology of VS infections in Norway and to explore marine environmental factors and climate conditions as predictors for public health responses.

**Methods:**

We conducted a retrospective cross-sectional study of VS infections reported in Norway from 2014 to 2018. Epidemiological data were collected through a nationwide survey of public health microbiology laboratories. Environmental data, including seawater temperature (SWT), salinity (SWS), atmospheric temperature (AT), and rainfall (RF), were obtained from Norwegian public monitoring systems. Negative binomial regression adapted for time-series data was used to estimate the short-term effect of marine environmental factors and climate conditions on the number of VS cases.

**Results:**

A total of 303 VS infections were reported with most cases (63%) occurring in the Southeast region of Norway. SWT, SWS, and AT showed significant correlations with VS cases, with a gradual non-linear increase in VS risk for seawater temperature above 13°C [RR 1.60; CI(95%):1.02,2.8]. A 1-month lag effect was observed with increased SWT and AT predicting VS cases. No significant association was found for RF at the national level, but regional differences were observed.

**Conclusions:**

VS infections in Norway are influenced by marine environmental factors and climate conditions. Validation of existing real-time models adapted to the regional conditions could enhance early public health responses to inform preventive measures for the at-risk population in Norway.

**Supplementary Information:**

The online version contains supplementary material available at 10.1186/s12889-026-27550-7.

## Background

*Vibrio* and *Shewanella* spp. (VS) are both environmental and climate-sensitive bacteria naturally found in marine and estuarine systems [1–3]. Approximately a dozen *Vibrio spp*. that cause vibriosis are of clinical relevance to humans, with *V. parahaemolyticus*, and *V. vulnificus* being among the most pathogenic species [4, 5], while within the genus *Shewanella*, only two species—*S. putrefaciens* and *S. algae—*are considered pathogenic to humans [3, 6]. Most VS infections are associated with mild illness such as gastroenteritis and otitis; however, some VS infections can lead to severe and life-threatening outcomes, such as sepsis or necrotizing fasciitis [4–6]. VS infections are mostly acquired through recreational exposure to seawater or the consumption of contaminated seafood [5, 7].

Although the highest number of VS infections has been usually reported in places with hot summers in tropical and sub-tropical regions [3, 8], an increasing numbers of VS infections have been more recently reported in temperate countries at high latitudes [9], particularly in the Northern European region over the past decades [4, 6]. Similar to other climate-sensitive illnesses, VS infections exhibit a distinct seasonal pattern, with most infections occurring during the summer months when seawater temperatures and salinity levels are optimal for bacterial growth (> 15 °C and < 25 ppt NaCl) [9, 10]. For example, several studies have shown that heatwaves, which lead to an increase in sea surface temperature (SST), are linked to a rise in the number of reported VS cases, particularly over the last decades in Nordic countries bordering the Baltic Sea [4, 6, 11, 12]. Furthermore, during the exceptionally warm summer of 2018, a cluster of septicaemia and necrotizing fasciitis caused by VS following exposure to high seawater temperatures in Southeast Norway was reported [4, 13]. Although temperature is a key factor for VS species, both temperature and salinity are generally strong and predictive correlates of their distribution and activity, particularly in brackish water environments [9, 14, 15].

Predicting changes in the occurrence of these infections, for example through early-warning systems, can facilitate early public health responses. For this purpose, the ECDC Geoportal *Vibrio* Map Viewer (https://geoportal.ecdc.europa.eu/vibriomapviewer/) examines environmentally suitable conditions such as sea surface temperature and salinity for *Vibrio* growth [9]. However, this model has been calibrated to the Baltic Region only and might not apply to other settings, such as the Norwegian coastline, without prior validation. Additionally, the model has not been calibrated for the risk of *Shewanella spp*. Furthermore, there is a knowledge gap regarding the influence of other exposure factors such as climate conditions (e.g., atmospheric temperature and rainfall) on the occurrence of VS infections.

The aim of this study was to describe the epidemiology of VS infections in Norway and to explore both marine environmental factors (seawater temperature and salinity) and climate conditions (atmospheric temperature and rainfall) as predictors for early warning purposes, timely response and preventive measures for these infections.

## Methods

### Study design

We conducted a retrospective cross-sectional study to investigate the epidemiology of VS infections reported in Norway during the 5-year period from January 2014 to December 2018. We evaluated the effect of both marine environmental factors and climate conditions, including seawater temperature (SWT), seawater salinity (SWS), atmospheric temperature (AT), and rainfall (RF), on short-term (monthly) reported VS infections both at national and regional level (Supplementary Figure S1). A case was defined as a laboratory confirmed *Vibrio* or *Shewanella* infection reported to a Norwegian microbiology laboratory during the study period. VS species identification was performed using MALDI-TOF. VS cases reporting travel history abroad were excluded from further analysis.

### Epidemiological and clinical data

We collected data on VS infections using a nationwide survey of Norwegian public health microbiology laboratories. The reported data included information on sex, age, and place of residence. Clinical information, such as sample material, type of infection, underlaying conditions, and hospitalization, were also collected. If date of symptom onset was not reported, the registration date was used as a proxy for the month and year of infection.

### Environmental data

Marine environmental data on SWT and SWS (monthly mean, median, minimum, and maximum) were obtained from FerryBox systems in operation on ships of opportunity (https://www.niva.no/en/ferrybox) developed by the Norwegian Institute of Water Research (NIVA) [16]. These data were collected through sensors (Seabird SBE38 and SBE45) installed at ~ 4 m depth aboard ferries travelling along the Norwegian coastline in four GPS locations as proxies for the North (69.72 deg N, 19.00 deg E), Centre (63.48 deg N, 10.21 deg E), West (60.48 deg N, 5.24 deg E) and Southeast (59.90 deg N, 10.69 deg E) regions. Climate data on AT and RF (monthly mean) were obtained from the Norwegian service for weather warnings and other meteorological information – Yr (https://www.yr.no/en). Data completeness was > 90% for all marine environmental variables and air temperature and > 80% for rain fall. Imputation of missing values was performed using the R package “imputeTS” and selecting the linear interpolation method.

### Epidemiological and statistical analysis

We described the epidemiology of VS cases reported in Norway over the study period. Data presented included the average incidence rate per 100,000 inhabitants, sex ratio, median age, distribution of cases across age groups, type of infections, hospitalisation, region, season, and year. For all marine environmental and climate variables, descriptive statistics were calculated (Supplementary Table S1), and correlation coefficients between each variable and the number of VS cases per study region were estimated. Within each set of marine environmental factors (mean, median, maximum and minimum) and climate conditions (mean), we selected the variables with highest correlation with the number of VS cases results for further model building.

### Modelling analysis

Correlation analyses were performed between each marine environmental factors, climate conditions, and the number of cases over the study period. A generalized linear model (GLM) with a Poisson distribution was used to study the short-term effects of marine environmental factors and climate conditions on VS occurrence, accounting for seasonality, trend, autocorrelation, region, and lagged effects. To compare the results from the four study regions, regression analysis was applied to data from each region separately. As overdispersion was observed in the outcome variable, a negative binomial approach was chosen. We accounted for seasonal and yearly variation by including a natural spline for time with up to 4 degrees of freedom per calendar year in the regression model (*Supplementary Figure S2*). Because the effect of marine environmental factors and climate conditions on disease occurrence could be delayed and/or last several weeks, we fitted the regression models with environmental data with time lags of 0, 1, 2 months. Lag structures were pre-specified based on ecological and epidemiological evidence as well as preliminary analyses. In addition, to account for the cumulative lag effect of environmental data with cumulative time lag (0–1 and 0–2 months) were considered in the final model. To explore the shape of the relationship between marine environmental factors and number of VS cases, natural splines with up to 4 degrees of freedom were fitted in the final model. We accounted for autocorrelation by incorporating into the models an autoregressive term at order 1 (VS cases lagged 1 month).

The model was built in a stepwise fashion by first constructing the long-term trend and seasonality model, then adding the marine and climate variable of interest. Each environmental exposure considered independently. Akaike’s Information Criterion (AIC) was employed to inform model specification for each environmental exposure, specifically to compare alternative lag structures (e.g., 0, 1, 2, and cumulative lags) and to determine the appropriate degrees of freedom for spline functions.

Plots of model residuals predicted and observed time-series plots, and partial autocorrelation function of the residuals were evaluated to ensure an adequate fit of the data. The results of the final model are expressed as changes in relative risk (RR) of monthly VS cases per 1 unit change in the explanatory variable. All analyses were performed using R Statistical Software (v4.3.0; R Core Team 2023, https://www.R-project.org/).

## Results

### Descriptive epidemiology of VS infections

A total of 303 domestic VS infections were reported in Norway during the 5-year study period, 2014–2018. The general epidemiological characteristics included information on average yearly incidence, sex, age group, type of infection, hospitalisation, region, season, and year of infection (Table 1).

**Table 1:** Summary of epidemiological characteristics of *Vibrio* and *Shewanella* cases in Norway, 2014–2018

**Characteristic**	***Shewanella***, *N* = 76^a^		***Vibrio***, *N* = 227^a^		***Overall***, *N* = 303^a^	
**Annual incidence/100,000 (SD)**	0.29	(0.18)	0.87	(0.47)	1.16	(0.66)
Sex						
Female	24	(32%)	130	(57%)	154	(51%)
Male	52	(68%)	97	(43%)	149	(49%)
Age	48	(13, 69)	31	(13, 60)	36	(13, 63)
Age group (years)						
0–4	1	(1.3%)	3	(1.3%)	4	(1.3%)
5–14	20	(26%)	65	(29%)	85	(28%)
15–24	6	(7.9%)	32	(14%)	38	(13%)
25–44	9	(12%)	40	(18%)	49	(16%)
45–64	12	(16%)	43	(19%)	55	(18%)
65–79	23	(30%)	32	(14%)	55	(18%)
80 +	5	(6.6%)	12	(5.3%)	17	(5.6%)
Type of Infection						
Blood	5	(6.6%)	5	(2.2%)	10	(3.3%)
Gastrointestinal	5	(6.6%)	7	(3.1%)	12	(4.0%)
Ear	27	(36%)	143	(63%)	170	(56%)
Wound	25	(33%)	58	(26%)	83	(27%)
Other	14	(18%)	14	(6.2%)	28	(9.2%)
Hospitalized	25	(33%)	24	(11%)	49	(16%)
Region						
Centre	7	(9.2%)	18	(7.9%)	25	(8.3%)
North	5	(6.6%)	10	(4.4%)	15	(5.0%)
Southeast	41	(54%)	150	(66%)	191	(63%)
West	23	(30%)	49	(22%)	72	(24%)
Season						
Autumn	26	(34%)	73	(32%)	99	(33%)
Spring	7	(9.2%)	11	(4.8%)	18	(5.9%)
Summer	38	(50%)	122	(54%)	160	(53%)
Winter	5	(6.6%)	21	(9.3%)	26	(8.6%)
Year						
2014	13	(17%)	39	(17%)	52	(17%)
2015	11	(14%)	34	(15%)	45	(15%)
2016	10	(13%)	32	(14%)	42	(14%)
2017	9	(12%)	31	(14%)	40	(13%)
2018	33	(43%)	91	(40%)	124	(41%)

*SD* Standard deviation

^a^n (%), Median (IQR)

When comparing the epidemiological characteristics of VS infections, we observed a similar pattern in terms of percentages of VS cases per region (Southeast), season (summer) and year (2018). Variations in characteristics were observed in relation to (i) sex, with higher percentages of males for *Shewanella* infections (68% vs. 43%), (ii) age groups, with higher percentages of infections in young adults (15–24 years old, 14% vs. 7.9%) and adult (25–44 years old, 18% vs. 12%) for *Vibrio*, and in the elderly (65–79 years old, 30% vs. 14%) for *Shewanella,* (iii) type of infection, with higher percentages of ear infections for *Vibrio* compared to *Shewanella* (63% vs. 36%), and (iv) hospitalisation, with a higher percentage of hospitalised cases for *Shewanella* infections compared to *Vibrio* infections (33% vs. 11%). For both *Vibrio* and *Shewanella* infections, the most common type of manifestation was ear infections (n = 170), followed by wound (n = 83), gastrointestinal (n = 12) and blood infections (n = 10). *V. alginolyticus* was the predominant *Vibrio* species for ear infections (n = 126, 88%). The highest number of wound infections per species were related to *V. parahaemolyticus* and *S. putrefaciens* (*n* = 13 and 17, respectively), while *V. vulnificus* was the species causing mostly blood and/or wound infections (*n* = 7) (*Supplementary Table S2*).

### Spatial–temporal distribution of VS infections, and their relationship with marine environmental factors and climate conditions

**Fig. 1:**
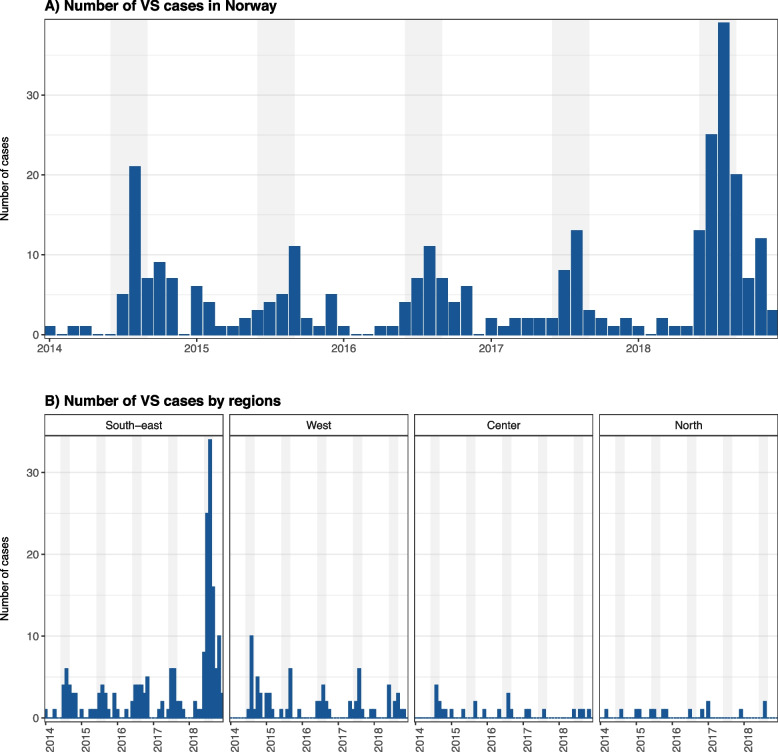
Total number of VS cases in Norway (**a**) and in each region (**b**) per month and year, Norway, 2014–2018

We observed a distinct seasonal pattern of domestic infections, with the highest number of VS cases reported during the summer months June–August (*n* = 160, 52.8%) over the 5-year study period in Norway. Two peaks of VS infections were observed during the summer months in 2014 and 2018. The main increase in number of VS cases was reported in August 2014 and was sustained over three months period in June–August 2018 (Fig. 1a). When stratifying by region, we observed a different distribution of VS cases, with the highest number of cases reported in the West region during the summer of 2014 (*n* = 13, 38.2%), and the Southeast region during the summer of 2018 (*n* = 71, 85.5%), (Fig. 1b). These findings are consistent with the above-average SWT recorded in the West region in July 2014 (19.5 °C) and prolonged above-average SWT in the Southeast region between June–August 2018 (19.4–21.3 °C) (*Supplementary Table S3*).

**Fig. 2:**
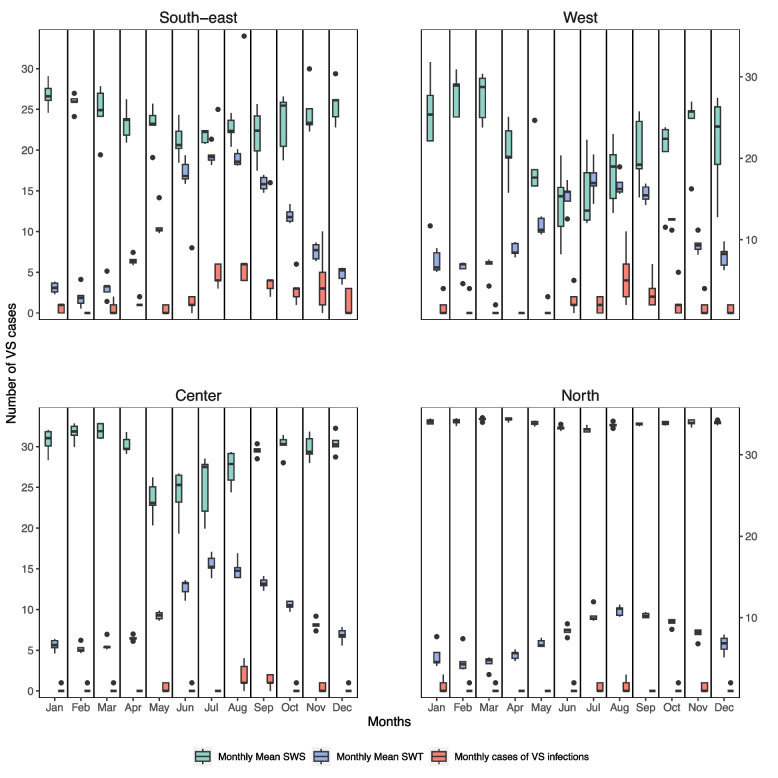
Range values for number of VS cases (in orange), SWT (in blue, expressed in °C) and SWS (in green, expressed in ppm) per month and region, Norway, 2014–2018

Overall, we observed marked seasonal variations in the number of VS cases, SWT and SWS. Higher numbers of VS cases, higher SWT, and lower SWS values during the summer months, June–August. During summers in the years 2014–2018, we observed different average maximum number of reported VS cases and monthly values ranges of SWT and SWS in the Southeast (*n* = 23.8; 15.9–21.3 °C; 18.5–24.5 PSU), West (*n* = 8.2; 11.6–19.5 °C; 7.2–22 PSU), Centre (*n* = 4.2; 11.1–17.1 °C; 19.3–29.3 PSU) and North (*n* = 1.4; 6.5–10.9 °C; 31.8–33.1 PSU) region (Fig. 2). AT followed a similar trend to SWT in each region (Southeast, 14.2–22.2 °C; West, 11.4–19.0 °C; Centre, 9.2–18.6 °C; North, 7.3–15.1 °C), while the West region was the only area with the broader range of monthly total RF (50.7–368.7 mm) during the summer months over the study period (*Supplementary Figure S3*).

### Association between VS cases, marine environmental factors and climate conditions

Correlation analysis showed that the number VS cases were overall significant and positively correlated with all SWT indicators (mean, maximum and minimum), as well as with mean AT (*Supplementary Figure S4*). Significant and negative correlations were observed between the number of VS cases and all SWS indicators, but no significant correlation was found between VS cases and monthly total RF (*Supplementary Figure S4*). Among marine environmental indicators, monthly mean and maximum of SWT and SWS showed the strongest correlation with the number of VS cases. Due to the high association amongst each set of marine environmental indicators (SWT and SWS) and to avoid multicollinearity, only monthly mean of SWT, SWS, AT and total RF were considered for further analysis.

**Fig. 3:**
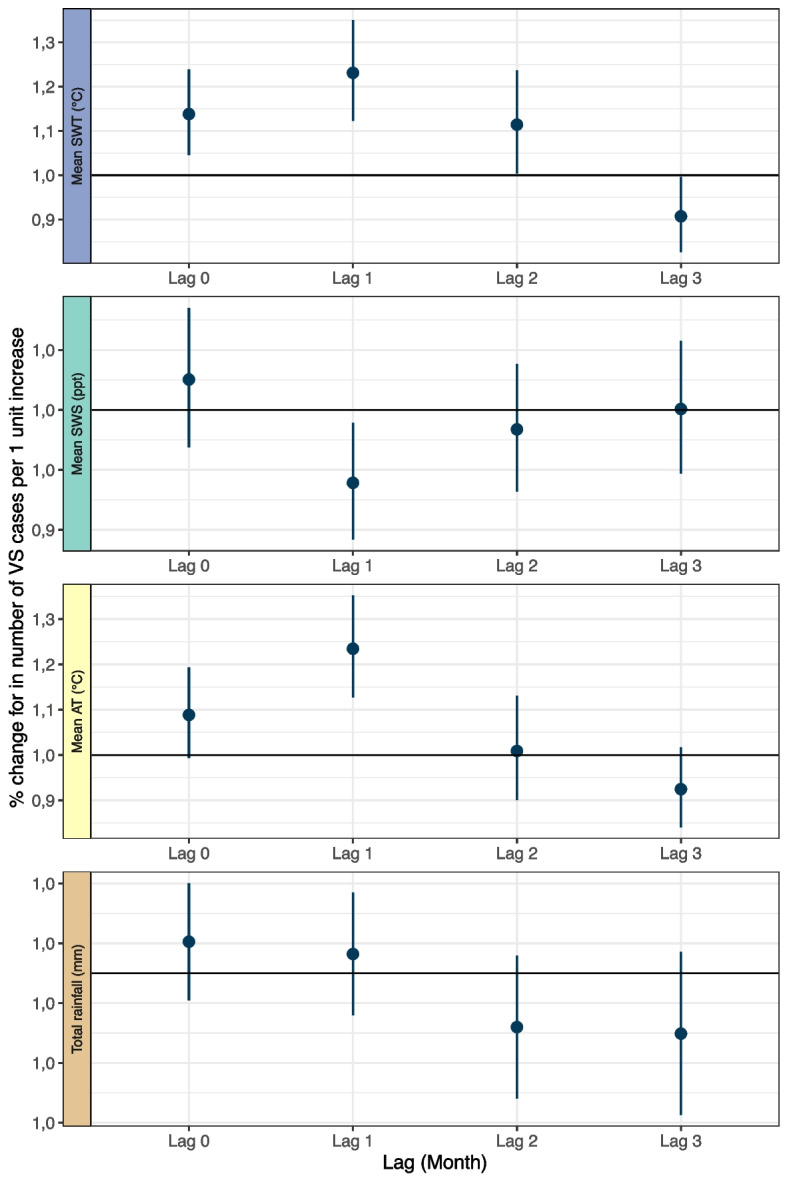
Overall lag effect of environmental and weather factors on number of VS cases in Norway, 2014–2018. Note: (i) Point estimates represent the per cent change in number of cases for 1 unit change in exposure. Bars reflect the 95% confidence interval

**Fig. 4:**
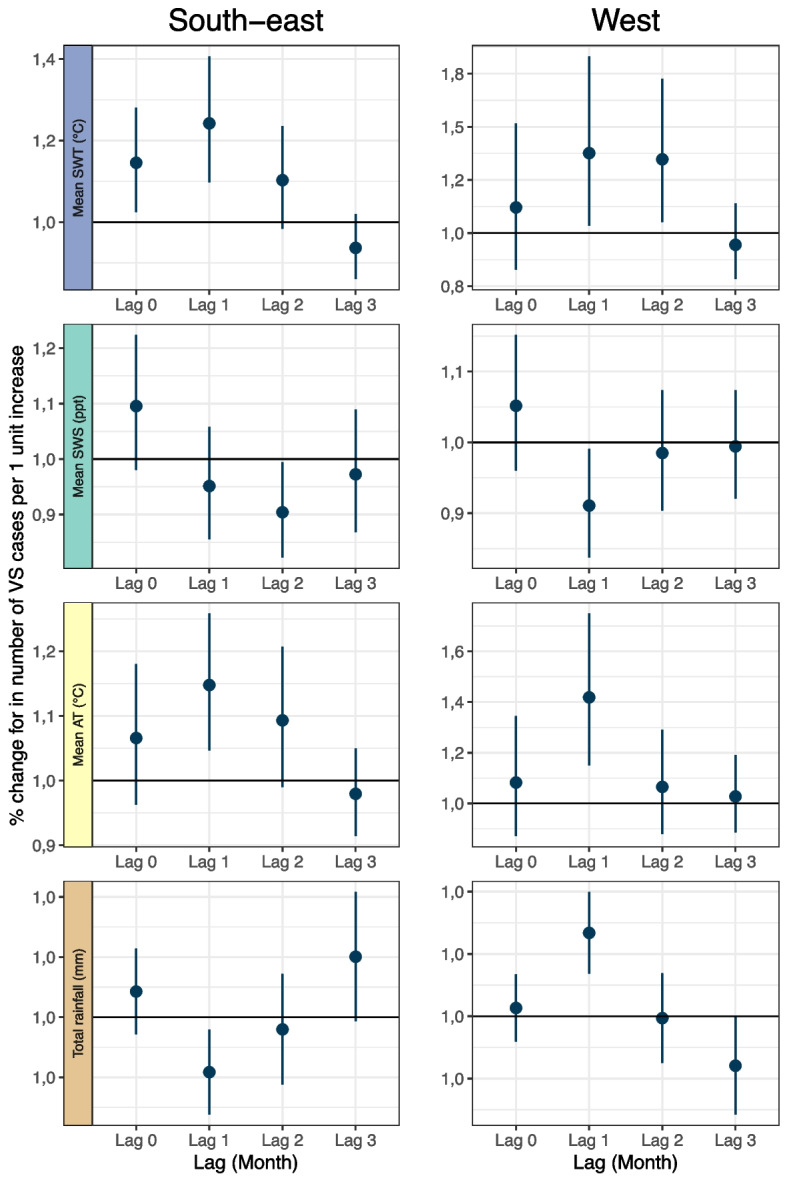
Region-specific lag effect of environmental and weather factors on number of VS cases Norway, 2014–2018. Note (i) Point estimates represent the per cent change in number of cases for 1 unit change in exposure. Bars reflect the 95% confidence interval

Negative binomial regression suggested that the number of VS cases was 1-order autoregressive and with significant seasonal and yearly distribution. After controlling for autocorrelation, seasonality, trend, and region, monthly mean SWT without lag and 1- and 2-month lag showed significant association with the number of VS cases (Fig. 3a). A negative significant association was observed between the number of VS cases and monthly mean SWS at 1-month lag (Fig. 3b), a positive significant association with mean AT without and at 1-month lag (Fig. 3c) and no association was observed for monthly total RF (Fig. 3d). The region-stratified regression analysis revealed significant associations between marine environmental factors and the numbers of VS cases reported in the Southeast and West regions. After controlling for seasonality, trend, and autocorrelation, monthly mean SWT with 1-month lag had the strongest association with VS cases only in these two regions. Monthly mean SWT without lag also showed association in the Southeast region but much less strongly (Fig. 4a). A similar effect was observed between AT and VS cases (Fig. 4c). Lag analyses indicated a negative association between VS cases and monthly mean SWS, at 1-month lag in the West region and at 2-month lag in the Southeast (Fig. 4b). Finally, total monthly RF with 1-month lag was negatively associated with VS cases in the Southeast and positively associated in the West region (Fig. 4d).

### Cumulative lagged effect of marine environmental factors and climate conditions

Based on the results obtained in the negative binomial regression analyses, two separated models were fitted to estimate the overall cumulative lagged effect of environmental and climate parameters on the occurrence of VS cases. Model 1 included each exposure variable with cumulative time lag (0–1 month) and model 2 included the exposure variable with cumulative time lag (0–2 months). All models were adjusted for seasonality, trend, autocorrelation, and regions.

**Fig. 5:**
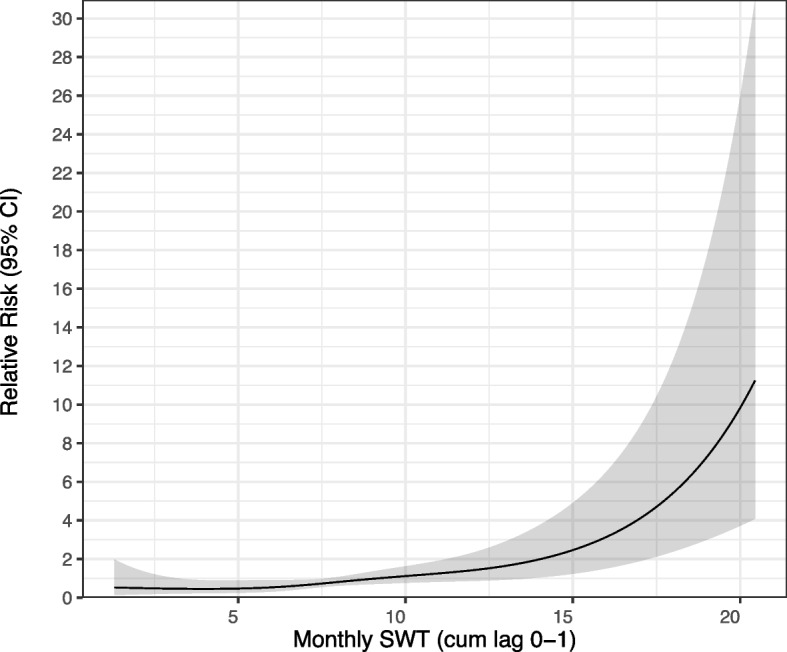
Overall relationship between the relative risk (RR) of VS infectious (monthly numbers of VS cases) and average seawater temperature (monthly mean) over lags 0–1 months (shown as a 4 d.f natural cubic spline), Norway, 2014–2018. Note (i) The relationship was adjusted for long-term trend and seasonal variation autocorrelation (AR-1) and region

For the overall cumulative lagged effect of SWT, the best fitted model was model 1 using a natural spline with four degrees of freedom. The selected model showed that the exposure–response relationship between VS cases and monthly mean SWT was mainly flat and not significant for SWT below 13 °C. Above this threshold, there was a significant positive association. At the threshold of 13 °C SWT, with a cumulative lag of 1 month, the relative risk (RR) was 1.60 [95% confidence interval (CI): 1.01, 2.91]. The estimated VS risk gradually increased successively beyond this threshold (Fig. 5).

Similar results were observed for the effect of AT on changes of proportion of VS cases (*Supplementary Figure S5*). For SWS and total RF, no significant results were identified when modelling the cumulative lagged effect.

## Discussion

Our study investigated the epidemiology of VS infections and the effect of marine environmental factors (SWT and SWS) and climate conditions (AT and RF) on the occurrence of VS infections in Norway during the 5-year study period from 2014 to 2018. Below we discuss: i) the epidemiology of VS infections, ii) the impact of environment and climate factors on VS infections, iii) their use as early-warning indicators, and iv) limitations and future research needs.

### Epidemiology of VS infections

We observed spatial–temporal similarities in the distribution of VS cases, with a higher percentage of infections reported in the Southeast Norway during summers (June–August), particularly in the warmer years of 2014 and 2018. These findings align with previous studies reporting a seasonal pattern and the influence of high seawater temperatures on these climate-sensitive bacteria [6, 11]. Differences in the epidemiological characteristics like sex, age groups, infection type, and hospitalisation could be related to (i) exposure of VS cases to different recreational activities (e.g., swimming, rowing, windsurfing, or fishing) by different demographic groups and, (ii) different pathogenicity, virulence and clinical manifestations by bacterial species [4, 6]. Additionally, *Shewanella* spp. are also known as spoilage microorganisms in marine fish, capable of causing gastrointestinal infections [17, 18]. Similar patterns in age and gender distribution for *Shewanella*-related infections have been previously reported [3]. As reported previously in a multi-country study in Northern Europe [4], the most common type of infections was otitis caused by *V. alginolyticus*. While *V. parahaemolyticus*, *V. vulnificus* and *S. putrefaciens* were the most frequently reported VS species causing severe infections (wounds and/or blood), consistent with previous studies highlighting their virulence [4, 6, 11, 13].

### Impact of environmental and climate on VS infections

From 2010 to 2020, global warming led to a ~ 0.28 °C rise in average ocean surface temperatures [19]. During this time, the longest sustained heatwave in Europe occurred between April and August 2018. Similarly, the surface water temperatures of the North Sea and the Baltic Sea in July 2018 were 2.0 °C and 2.8 °C above the average temperature in the twentieth century for July, respectively [20]. This warming trend was reflected in an increase of VS infections in the Northern Europe [4, 6, 11]. It is well established that the occurrence of VS infections is related to marine environmental conditions favourable for bacterial growth and correlates with seawater temperature above 18 °C and salinity below 25 PSU [21–23]. Our findings confirm this, with the highest number of VS cases reported during the exceptionally warm summer of 2018, especially in the Southeast and West region of Norway, where both seawater temperature and salinity were optimal for bacterial growth.

### Environmental factors and climate conditions as predictors for VS infections

At the national level, we observed a 1-month lagged effect for SWT, SWS and AT on the number of VS cases, consistent with other studies reporting a 2–5 weeks lagged effect of temperature on *Vibrio* infections [24, 25]. Both favourable marine environmental factors for bacterial growth and favourable climate conditions such as AT for recreational activities could contribute in human exposure, and therefore in the VS risk [26]. However, the 1-month lagged effect observed for SWT and AT suggests that marine environmental factors may better predict the number of VS cases observed in the following month. While we found no association between the number of VS cases and RF at the national level, we observed a marked geographic difference when stratifying by region, with a negative association in the Southeast and a positive association in the West region of Norway. We hypothesize that the following factors could have influenced these outcomes: (i) increased RF causing lower SWS levels and/or higher eutrophication due to the input of nutrients and organic matter run-off from rivers [16, 27, 28] in the West region, and (ii) decreased RF leading to higher human exposure through recreational activities and/or higher productivity by phytoplankton due to increased and prolonged light availability and therefore bacterial production [27, 28] in the Southeast region.

In our final model, the cumulative lagged effect of exposure–response relationship between mean SWT and VS cases was mainly flat and not significant for seawater temperature below 13 °C but became positively non-linear associated with VS cases above this threshold. The risk of VS infections increased with higher temperatures, aligning with previous studies linking rising coastal seawater temperatures to higher VS infection rates [6]. While the influence of temperature on *Vibrio* spp. growth is well-documented, the exact exposure–response relationship remains unclear. Baker-Austin et al. [9] reported a linear effect of SWT on the occurrence of vibriosis across all SST values. Similarly, Jacobs et al. [29] reported a linear increase of *V. vulnificus* abundance when SWT increased above the thresholds of 11 °C. Using a conditional logistic regression model, Semenza et al. [24] identified an exposure–response effect at SWT of 16 °C, and similar to our findings, the estimated effect of SST increased successively above this SST threshold. Sheahan et al. [30] developed three statistical models to evaluate the effect of SWT on three *Vibrio* spp. reporting a different effect of SWT for each *Vibrio* species. The different thresholds reported in the literature and in the present study could be explained by different methodologies applied, specific pathogenic species investigated, or different geographical areas monitored where other environmental factors or climate conditions, as shown in our study, likely play a role.

### Study limitations and future area of research

Our study has several limitations. When symptoms onset dates were unavailable, we used the registration date as a proxy for infection date. Cases without travel history were considered domestic, which may have overestimated local VS cases. Furthermore, the region of residence was used as proxy for region of infection. Mild infections such as otitis might have been reported with a delay or underreported. Environmental and climate data gaps were addressed using linear interpolation; however, as a single imputation method, this approach does not account for uncertainty in missing values and may therefore underestimate variability in subsequent analyses. While multiple imputation could provide a more robust framework by propagating this uncertainty, it was not implemented in the present study. Data were aggregated and analysed by region and month to increase statistical power. Given the low incidence of these infections, extending the study period could allow for increased temporal granularity (e.g., analysing weekly data), provide a deeper insight into the early warning window during both exceptionally warm and regular years, as well as testing separate models for VS infections. The models and findings from this study provide a proof-of concept for future studies, such as analysing data from the Norwegian Communicable Disease Reporting System (MSIS) to assess how the environmental and climate conditions affect the VS public health risk along the Norwegian coastline over time. Given the small sample size for this analysis, we focused only on estimating the main effects of each marine and climate variables separately, without examining combined and/or interactive effects. In addition, the restriction to lag periods (0–2 months) may have limited our ability to detect longer-term delayed effects, although this choice was made to ensure model stability given the low incidence of cases. More flexible approaches, such as distributed lag models, could be explored in future studies with higher temporal resolution and larger datasets to more comprehensively characterise lagged associations. Based on our study design and aim, we cannot conclude on other ecological phenomena which could have played a role or being part of an ecological succession. Investigating additional environmental parameters may provide more insights in these aspects. Therefore, future interdisciplinary studies are needed to reach final conclusions.

## Conclusions

VS are environmental, climate-sensitive bacteria that cause seasonal waterborne diseases. Following the VS outbreak in 2018 and the global increase in seawater temperatures, VS infections were made mandatory notifiable diseases to MSIS in June 2019, despite their overall low occurrence in Norway. Both environmental factors and climate conditions influence the occurrence of VS infections, particularly at the regional level. Considering that the ECDC Geoportal Vibrio map viewer has been calibrated only to the Baltic region, we recommend validating this real-time model tool for the Norwegian coastline using a non-linear model with a seawater temperature threshold above 13 °C to examine the favorable environmental conditions for bacterial growth. If the purpose of the model is to be used as an early warning system and to estimate the risk of VS infections, a 1-month lag effect on seawater temperature and salinity – particularly during sustained heatwaves – along with the inclusion of real-time climate parameters such as atmospheric temperature and total rainfall, should be considered in order to improve the accuracy, reliability, timeliness of the information produced for public health response.

## Supplementary Information

Supplementary Material 1.

## Data Availability

The datasets generated and/or analysed during the current study are not publicly available due legal restrictions related to data protection but are available from the corresponding author on reasonable request.

## Abbreviations

VS: Vibrio and Shewanella species
NIPH: Norwegian Institute of Public Health
EPIET: ECDC Fellowship Programme, Field Epidemiology path
ECDC: European Centre for Disease Prevention and Control
NIVA: Norwegian Institute for Water Reasearch
SWT: Seawater Temperature
SWS: Seawater salinity
AT: Atmospheric temperature
RF: Rainfall
RR: Relative Risk
CI: Confidence Interval
SST: Sea Surface Temperature
GLM: Generalized Linear Model
AIC: Akaike Information Criteria
IQR: Interquartile Range
SD: Standard Deviation
PSU: Practical Salinity Unit
MSIS: The Norwegian surveillance system for communicable diseases
