# Supplementary material for: Impact of environmental factors and climate conditions on the occurrence of Vibrio and Shewanella infections in Norway, 2014–2018
Source: BMC Public Health. 2026 May 4;26:1942. doi: 10.1186/s12889-026-27550-7 (PMC13285024; doi:10.1186/s12889-026-27550-7)
Supplement: Supplementary file 1 — Supplementary Material 1. [file 12889_2026_27550_MOESM1_ESM.docx]

**SUPPLEMENTARY MATERIAL**

**TABLES**

***Table S1.*** Descriptive statistics of values and range of environmental factors (seawater temperature and salinity) and climate conditions (atmospheric temperature and total rainfall) by region in Norway, 2014-2018.

|  | South-East | West | Centre | North |
| --- | --- | --- | --- | --- |
| **Mean SWT**  **(range)** | 10.0  (0.5, 21.3) | 10.2  (3.3, 19.5) | 9.5  (4.6, 17.1) | 6.5  (2.0, 10.9) |
| **Minimum SWT**  **(range)** | 7.9  (-0.8, 19.1) | 9.0  (2.8, 18.0) | 8.1  (2.5, 14.9) | 5.8  (1.3, 10.1) |
| **Maximum SWT**  **(range)** | 11.9  (1.7, 23.1) | 11.5  (3.9, 20.8) | 10.8  (5.4, 20.4) | 7.4  (3.1, 12.2) |
| **Mean SWS**  **(range)** | 23.8  (17.5, 30) | 20.0  (7.2, 30.8) | 28.7  (19.3, 32.9) | 32.9  (31.8, 33.6) |
| **Minimum SWS**  **(range)** | 22  (16, 29) | 16  (3, 29) | 26  (10, 33) | 32  (29, 33) |
| **Maximum SWS**  **(range)** | 25.3  (19.1, 31.4) | 25.0  (11.0, 33.2) | 30.9  (24.3, 33.4) | 33.2  (32.7, 33.8) |
| **Mean AT**  **(range)** | 8.0  (-4.4, 21.2) | 8.9  (0.0, 19.0) | 5.6  (-5.1, 18.6) | 3.8  (-5.6, 15.1) |
| **Mean RF**  **(range)** | 101  (10, 273) | 224  (51, 540) | 82  (3, 240) | 82  (2, 236) |

*Note:* Seawater temperature (SWT); Seawater salinity (SWS); Atmospheric temperature (AT); total rainfall (RF). The monthly mean SWT in Norway ranged from 6.5 °C in the northern part of Norway to 10.2 in the Western region, with a minimum of -0.8°C in 2017 and maximum of 23°C during 2018 in the Southeastern region. For all four study regions, both mean and maximum monthly seawater temperatures were highest during July for the entire study period. The mean seawater salinity ranged 20-28.7 PSU with a minimum of 3 PSU in the western region during 2015 and maximum of 33.8 in the Northern region during 2018.

***Table S2.*** Distribution of VS cases per species and type of infection, Norway 2014-2018

|  | Type of manifestation or site of infection, no. (%) | | | | |
| --- | --- | --- | --- | --- | --- |
| VS species | Ear  **(**N=170**)** | Wound  **(**N=83**)** | GI^1^  **(**N=12**)** | Blood  **(**N=10**)** | Other  **(**N=28**)** |
| *Shewanella putrefaciens* | 23 | 17 | 5 | 4 | 10 |
| *Shewanella algae* | 4 | 3 | 0 | 1 | 4 |
| *Shewanella profunda* | 0 | 1 | 0 | 0 | 0 |
| *Shewanella* spp. | 0 | 4 | 0 | 0 | 0 |
| *Shewanella* Total | **27 (16%)** | **25 (30%)** | **5 (42%)** | **5 (50%)** | **14 (50%)** |
| *Vibrio alginolyticus* | 126 | 40 | 3 | 0 | 8 |
| *Vibrio parahaemolyticus* | 6 | 13 | 2 | 1 | 2 |
| *Vibrio vulnificus* | 2 | 3 | 0 | 4 | 1 |
| *Vibrio cholerae* NT ^2^ | 2 | 0 | 2 | 0 | 0 |
| *Vibrio fluvialis* | 0 | 0 | 0 | 0 | 1 |
| *Vibrio* spp. | 7 | 2 | 0 | 0 | 2 |
| *Vibrio* Total | **143 (84%)** | **58 (70%)** | **7 (58%)** | **5 (50%)** | **14 (50%)** |

*Note^1^:* Gastrointestinal infections; *Note^2^:* Non-toxigenic *Vibrio cholerae*.

***Table S3.*** Seawater temperature (°C) recorded in Southeast region (Oslo) and West region (Bergen) per month and year, Norway, 2014-2018.

| **Year** | **Month** | | | | | | | | | | | |
| --- | --- | --- | --- | --- | --- | --- | --- | --- | --- | --- | --- | --- |
|  | **Jan** | **Feb** | **Mar** | **Apr** | **May** | **Jun** | **Jul** | **Aug** | **Sep** | **Oct** | **Nov** | **Dec** |
| **Average seawater temperature (°C) ‘Southeast region’** | | | | | | | | | | | | |
| **2014** | 3.8 | 2.2 | 3.3 | 6.6 | 10.2 | 18.2 | **-** | 20.1 | 16.6 | 13.4 | 8.7 | 5.2 |
| **2015** | 3.7 | 4.1 | 5.1 | 7.4 | 10.4 | 15.9 | 18.8 | 18.1 | 14.8 | 12.4 | 8.4 | 5.6 |
| **2016** | 2.6 | 1.9 | 3.3 | 6.2 | 10.1 | 16.8 | 19.4 | 18.6 | 17.0 | 11.3 | 6.6 | 3.5 |
| **2017** | 2.3 | 1.2 | 1.4 | 5.9 | 9.8 | 16.5 | 18.2 | 18.3 | 15.3 | 11.1 | 6.4 | 4.3 |
| **2018** | 3.1 | 0.5 | 2.6 | 6.4 | 14.2 | 19.4 | 21.3 | 19.6 | 15.8 | 11.8 | 7.7 | 5.4 |
| **Average seawater temperature (°C) ‘West region’** | | | | | | | | | | | | |
| **2014** | 7.9 | 5.4 | 6.0 | 8.7 | 11.6 | 16.3 | 19.5 | 17.9 | 15.9 | 11.6 | - | - |
| **2015** | - | 6.0 | 6.6 | 7.3 | 10.2 | 11.6 | 13.4 | 14.9 | - | 11.7 | 8.3 | 5.3 |
| **2016** | 5.1 | 6.2 | 6.2 | 7.4 | 11.9 | - | 15.6 | 14.7 | 15.5 | 11.4 | 7.2 | 7.3 |
| **2017** | 5.6 | 6.0 | 6.3 | 6.8 | 9.7 | 14.9 | 15.9 | 15.2 | 14.5 | 11.5 | 8.6 | 5.9 |
| **2018** | 5.3 | 3.6 | 3.3 | 8.5 | 9.9 | 14.9 | 17.2 | 16.0 | 14.0 | 10.2 | 7.8 | 7.5 |

**FIGURES**


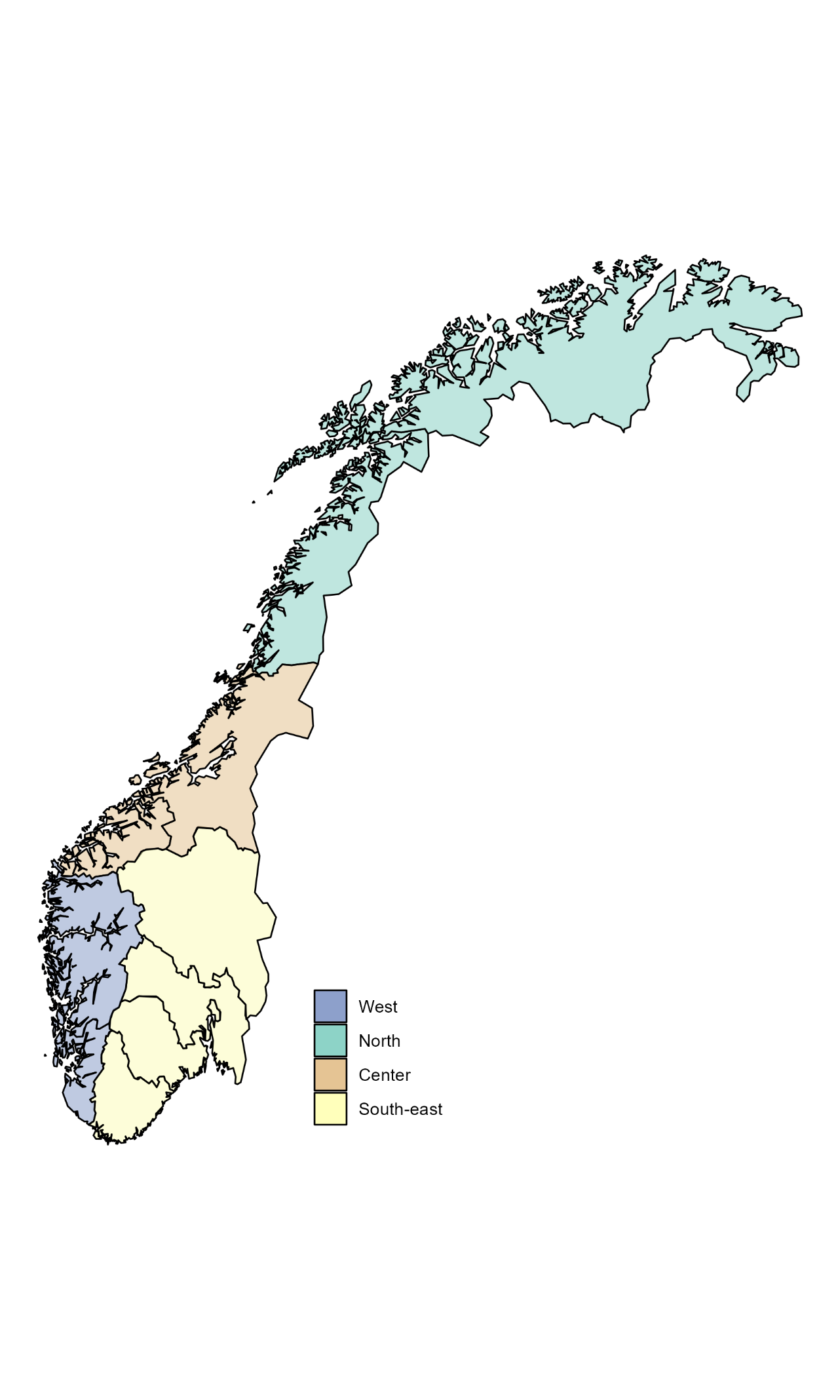


***Figure S1.*** Geographical map of Norway. The colours indicate the four health and administrative regions.


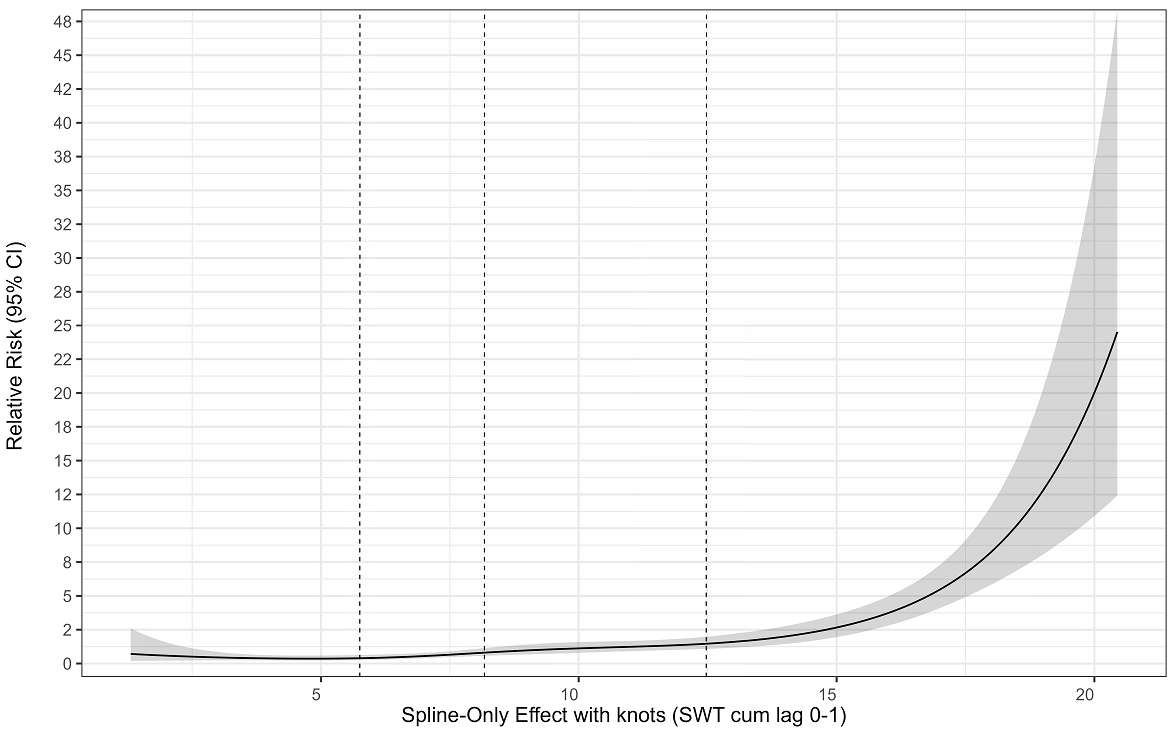


***Figure S2.*** Association between temperature and relative risk modeled using natural cubic splines (4 degrees of freedom) in a quasi-Poisson regression without adjustment for additional covariates. Estimates are presented relative to the median temperature. Shaded areas represent 95% confidence intervals. Vertical dashed lines indicate knot locations.


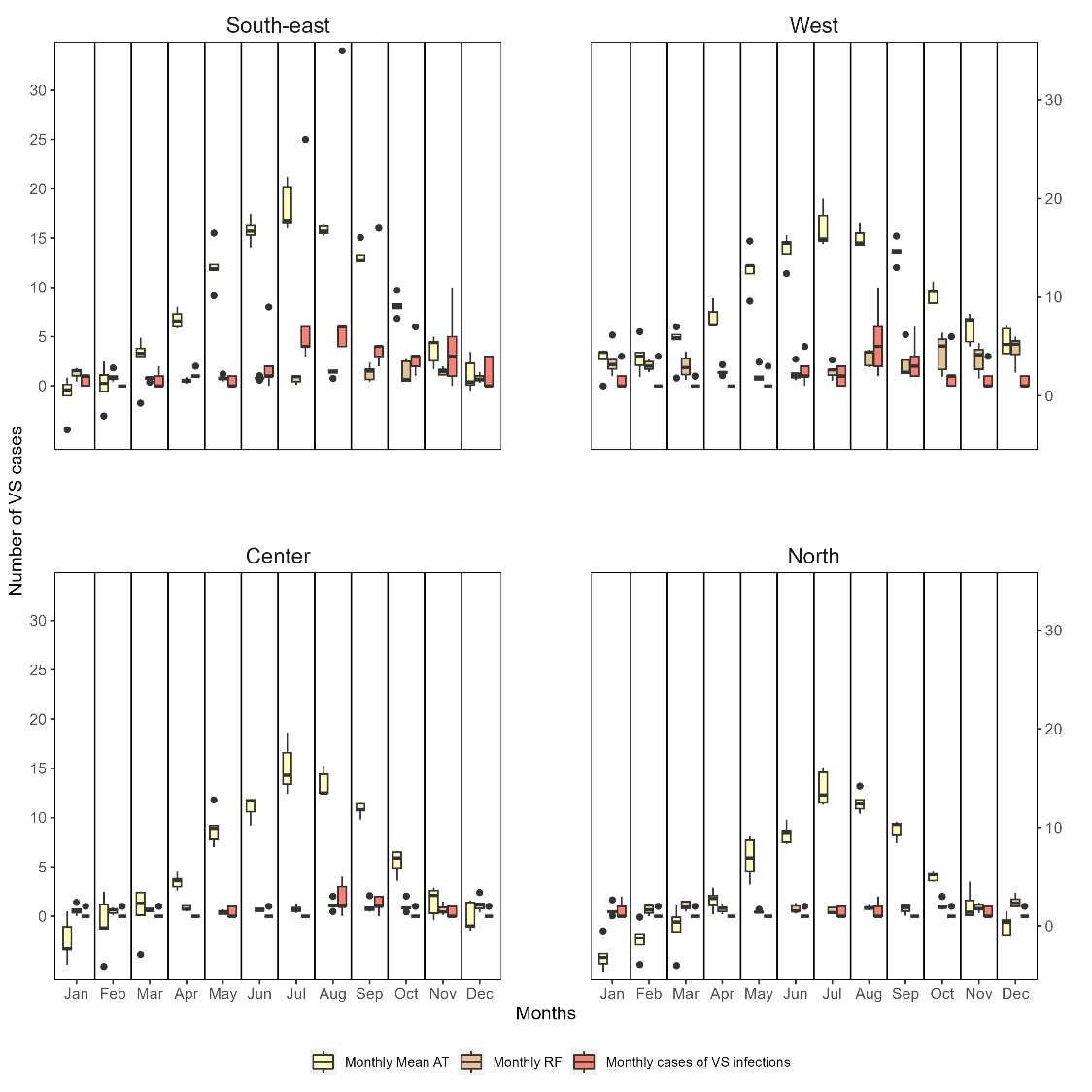


***Figure S3.*** Range values for number of VS cases (in orange), AT (in yellow, expressed in °C, right y-axis) and RF (in brown, expressed in PSU, right y-axis) per month and region, Norway, 2014-2018.


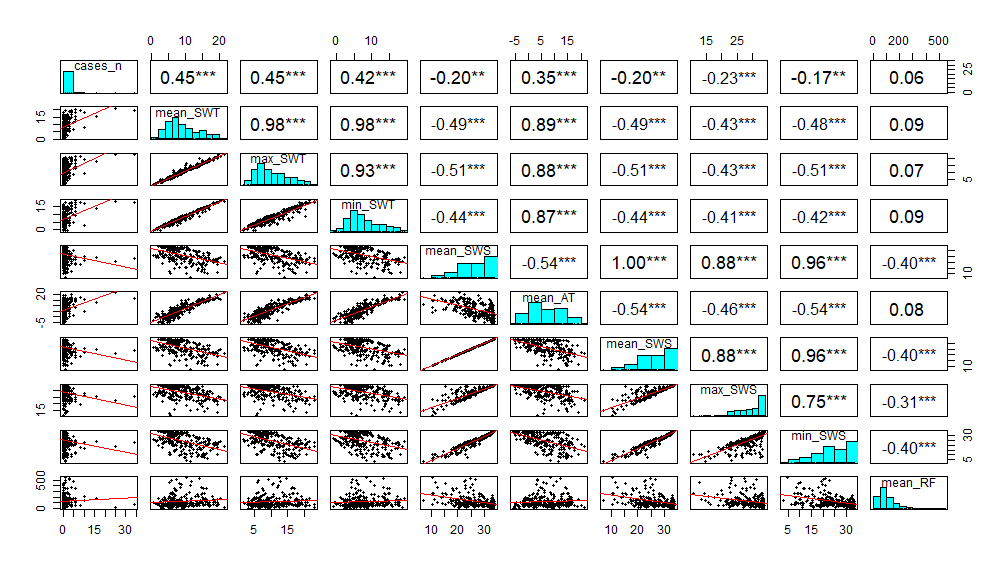


***Figure S4.*** *Correlation coefficient matrix, scatter plot, and frequency distribution for study variables. Each variable's distribution is displayed the diagonal. The bivariate scatter plots with a trend line are shown at the bottom of the diagonal. The correlation coefficient and the level of significance are displayed as stars at the top of the diagonal * p ≤ 0.05, ** p ≤ 0.01, and *** p > 0.001 show significance level.*

***Note:*** *Cases_n, monthly VS cases, mean_SWT, monthly mean seawater temperature; max_SWT, monthly maximum seawater temperature; min_SWT , monthly minimum seawater temperature; mean_SWS, monthly mean seawater salinity; max_SWS, monthly maximum seawater salinity; min_SWS , monthly minimum seawater salinity, mean_AT, monthly mean air temperature; mean_RF, monthly rain fall.*

***
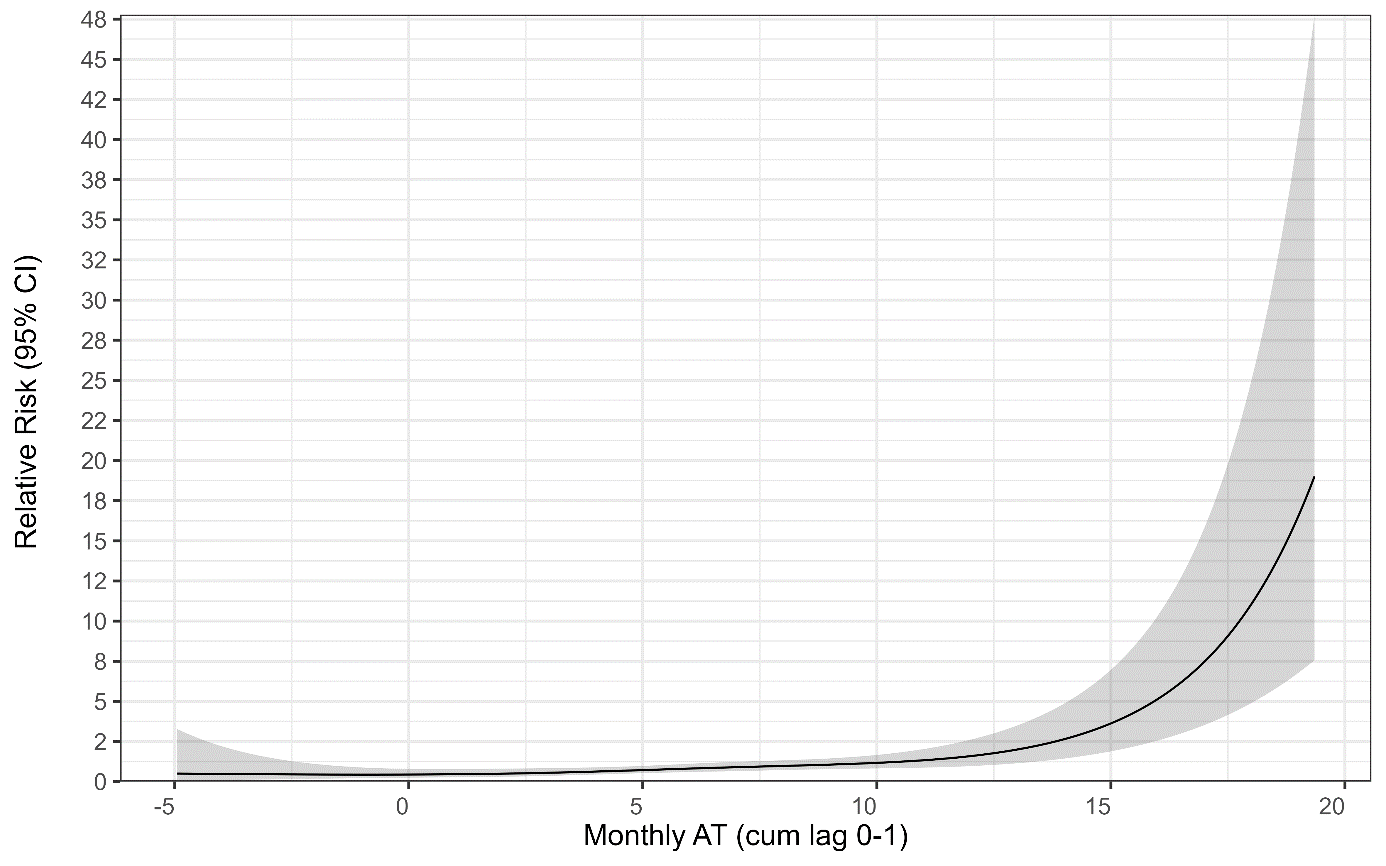
***

***Figure S5.*** The overall relationship between the relative risk (RR) of VS infectious (monthly numbers of VS cases) and atmospheric temperature (monthly mean) over lags 0-1 months (shown as a 4 d.f natural cubic spline). The relationship was adjusted for long-term trend and seasonal variation autocorrelation and region, Norway, 2014-2018.
